# The Dermatan Sulfate Proteoglycan Decorin Modulates α2β1 Integrin and the Vimentin Intermediate Filament System during Collagen Synthesis

**DOI:** 10.1371/journal.pone.0050809

**Published:** 2012-12-03

**Authors:** Oliver Jungmann, Katerina Nikolovska, Christian Stock, Jan-Niklas Schulz, Beate Eckes, Christoph Riethmüller, Rick T. Owens, Renato V. Iozzo, Daniela G. Seidler

**Affiliations:** 1 Insitute of Physiological Chemistry and Pathobiochemistry, University Hospital Münster, Münster, Germany; 2 Institute of Physiology II, University Hospital Münster, Münster, Germany; 3 Department of Dermatology, University of Cologne, Cologne, Germany; 4 Center for Nanotechnology, Serend-ip GmbH, Münster, Germany; 5 LifeCell Corporation, Branchburg, New Jersey, United States of America; 6 Department of Pathology, Anatomy and Cell Biology, Thomas Jefferson University, Philadelphia, Pennsylvania, United States of America; Lerner Research Institute, United States of America

## Abstract

Decorin, a small leucine-rich proteoglycan harboring a dermatan sulfate chain at its N-terminus, is involved in regulating matrix organization and cell signaling. Loss of the dermatan sulfate of decorin leads to an Ehlers-Danlos syndrome characterized by delayed wound healing. Decorin-null (Dcn^−/−^) mice display a phenotype similar to that of EDS patients. The fibrillar collagen phenotype of Dcn^−/−^ mice could be rescued in vitro by decorin but not with decorin lacking the glycosaminoglycan chain. We utilized a 3D cell culture model to investigate the impact of the altered extracellular matrix on Dcn^−/−^ fibroblasts. Using 2D gel electrophoresis followed by mass spectrometry, we identified vimentin as one of the proteins that was differentially upregulated by the presence of decorin. We discovered that a decorin-deficient matrix leads to abnormal nuclear morphology in the Dcn^−/−^ fibroblasts. This phenotype could be rescued by the decorin proteoglycan but less efficiently by the decorin protein core. Decorin treatment led to a significant reduction of the α2β1 integrin at day 6 in Dcn^−/−^ fibroblasts, whereas the protein core had no effect on β1. Interestingly, only the decorin core induced mRNA synthesis, phosphorylation and de novo synthesis of vimentin indicating that the proteoglycan decorin in the extracellular matrix stabilizes the vimentin intermediate filament system. We could support these results in vivo, because the dermis of wild-type mice have more vimentin and less β1 integrin compared to Dcn^−/−^. Furthermore, the α2β1 null fibroblasts also showed a reduced amount of vimentin compared to wild-type. These data show for the first time that decorin has an impact on the biology of α2β1 integrin and the vimentin intermediate filament system. Moreover, our findings provide a mechanistic explanation for the reported defects in wound healing associated with the Dcn^−/−^ phenotype.

## Introduction

Decorin belongs to the small leucine-rich proteoglycans and is covalently linked with a linear glycosaminoglycan (GAG) chain. Depending on the tissue the GAG chain is either chondroitin or dermatan sulfate (CS/DS). CS is composed of disaccharide repeats of D-glucuronic acid (GlcA) and *N*-acetylgalactosamine (GalNAc). The stereochemistry of the glycosidic linkage in CS is (βD-GlcA1’->3′ βD-GalNAc1’->4′-). In DS the D-GlcA is epimerized to l-iduronic acid (l-IdoA) with a glycosidic stereochemistry (αl-IdoA1’->3′ βD-GalNAc1’->4′-). The function of decorin is diverse and ranges from modulating collagen fibrillogenesis [1], [2] and matrix organization [3] to cell adhesion and migration [4]. Furthermore, decorin can act as a signaling molecule by binding to receptors such as the epidermal growth factor receptor [5], [6], [7], insulin-like growth factor receptor-1 [8], [9], cMET/hepatocyte growth factor receptors [10] or LDL receptor-related protein-1 (LRP1) [11]. In a contact allergy model the loss of decorin leads to a reduced edema formation and to an increase in sydecan-1 expression [12]. Also, decorin is able to decrease the amount of miR-21 and IL-10 and to stimulate the production of the proinflammatory tumor suppressor PDCD4 [13]. In addition, decorin can modulate transforming growth factor-β bioavailability [14].

The importance of the GAG chain of decorin was shown in EDS patients harboring mutations in the *βGALT4* gene which cause reduced enzymatic activity [15], [16]. Patients’ skin fibroblasts synthesized decorin partially without a GAG chain and the remaining GAG chains displayed reduced epimerization [16]. More recently, a new form of EDS was described. These patients exhibit only CS in the dermis due to the deficiency in the enzyme dermatan-4 sulfotransferase (*Chst*15) [17]. The loss of DS leads to a cutis laxa condition characterized by reduced deposition of collagen bundles in the dermis demonstrating the importance of DS in the dermal architecture [18]. An animal model with a genetic ablation of the dermatan C5 epimerase (EC 5.1.3.19) leads to only 20% l-IdoA in DS in the skin which shows an increased fibrillar diameter in the collagens [19]. The Dcn*^−/−^* mice display a skin EDS phenotype showing fibrils with an altered fibrillar diameter and abnormal supramolecular organization resulting in skin fragility [20] and delayed wound healing [21]. Using a 3D cell culture model of Dcn^−/−^ fibroblasts the fibrillar collagen phenotype was rescued by addition of decorin [22]. Interestingly, addition or viral expression of GAG-free decorin in Dcn^−/−^ cells induce a phenotype similar to that seen in the dermatan C5 epimerase^−/−^ mice [19] with an increased fibrillar diameter [23] indicating that the decorin GAG chain is important for regulating both shape and size of the collagen I fibrils. These examples show that not only GAGs but also the amount of epimerization of the GAG is important for matrix organization and dermal wound healing.

The collagen binding integrins α1β1, α2β1 and α11β1 are expressed on fibroblasts [24]. On a cellular level, Dcn^−/−^ fibroblasts show an increase in β1 integrin expression as compared to wild-type lung fibroblasts, and this leads to an enhanced adhesion to collagenous matrices [4]. Fibroblasts synthesizing their own 3D matrix use α5β1 integrin for adhesion, the major receptor for fibronectin [25]. Previously, it has been shown that decorin binding to β1 integrin requires the GAG chain [26]. Furthermore, only α2β1 but not α1β1 integrin is modulated by the proteoglycan decorin [27].

The expression pattern of intermediate filaments (IF) is cell and tissue specific [28], and fibroblasts contain the IF vimentin [29]. Vimentin belongs to the type III cytoplasmic IF type and shows a highly conserved secondary structure [30]. The IF system is a highly dynamic structure regulated by an equilibrium between subunits and polymers [31]. The IF vimentin is involved in the regulation of cell adhesion to collagens [32], [33]. In vitro studies show that vimentin can interact with α2β1 integrin cytoplasmic domains [34]. The main function of the IF vimentin is the maintenance of the cell and tissue integrity, cell shape and resistance to mechanical stress. Furthermore, it is involved in the intracellular distribution and function of organelles [35], [36]. Vimentin also contributes to the retrograde transport of Erk1/2 in injured neurons [37]. Vimentin^−/−^ mice undergo normal embryonic development. The mice appeared to develop and reproduce normally [38]. Vimentin-deficient fibroblasts display a reduced mechanical stability and aberrant focal adhesions [39]. Challenging these mice in vivo by wounding, vimentin-deficiency leads to a delay in wound healing due to reduced migration of fibroblasts [40].

The aim of our study was to determine the impact of both a Dcn^−/−^ matrix and a matrix deficient in the decorin dermatan sulfate chain on the vimentin IF system in fibroblasts. We discovered that the proteoglycan decorin is required for regulating β1 integrin levels in Dcn^−/−^ fibroblast in order to stabilize the vimentin IF system. The results obtained in the 3D cell culture system were confirmed *in vivo* in the dermis of Dcn^−/−^ mice compared to wild-type dermis. These results show that under matrix synthesizing conditions decorin is necessary for the vimentin IF system and that the mediating link is the α2β1 integrin. The present results provide a mechanistic explanation for the increased adhesion of Dcn^−/−^ fibroblasts and the delay in wound healing.

## Materials and Methods

### Decorin-null and Integrin α_2_-Null Mice

Dcn^−/−^ mice [20] were bred in the animal facility of the Institute of Physiological Chemistry and Pathobiochemistry of the University of Münster in accordance with the German Animal Protection Act (May 25^th^ 1998) and approved by LANUV, NRW, Germany. Mice deficient for the integrin α_2_ subunit and corresponding littermate controls were generated by heterozygous matings as previously described [41]. Mice were sacrificed with CO_2_ and all efforts were made to minimize suffering.

### Materials

The following primary antibodies were used: vimentin (monoclonal rabbit anti-mouse, Epitomics, California), vimentin (monoclonal rabbit anti-mouse, BioVision, San Francisco), phosphorylated vimentin pSer72 (monoclonal rabbit anti-mouse, Epitomics, California), type 1 collagen (rabbit anti-mouse, Acris, Herford, Germany), Ki67 (Dako), decorin LF113 (rabbit anti-mouse) [42], fibronectin (rabbit anti-mouse), polyclonal rabbit anti-mouse β1 integrin (Millipore) recognizing the cytosolic C-terminal domain, LEAF™ β1 integrin blocking antibody (armenian hamster anti-mouse, BioLegend, California), β1 integrin clone 9EG7 (BD Pharmingen) against the active conformation of the β1 integrin and α2 integrin (R&D), α5 integrin (polyclonal rabbit anti-mouse, Millipore), P-Tyr/Ser/Thr (Stressgen, MI, USA). Secondary antibodies were: Alexa 488 goat anti-rabbit, Alexa 488 goat anti-mouse (Molecular Probes, Eugene, Oregon) and Cy3 donkey anti-rat (Dianova) as well as donkey anti-rabbit IgG HRP (GE Healthcare, UK), donkey anti-sheep/goat IgG HRP (Millipore) and rabbit anti-mouse IgG HRP (Sigma). F-actin cytoskeleton was visualized by Alexa 488 labeled phalloidin (Lonza, Walkersville). DS was obtained by β-elimination from decorin derived from human skin fibroblasts [16].

### 3D Cultures of Primary Fibroblasts

Skin fibroblasts were obtained from one-day-old Dcn^−/−^ mice [20] or integrin α_2_^−/−^ mice [40] and respective controls. Cells were cultured in modified Eagle’s minimum essential medium (MEM) with Earle’s salts and supplemented with 10% fetal calf serum (Biochrom, Berlin, Germany), 2 mM glutamine and 100 U/ml penicillin–0.1 mg/ml streptomycin (PAA, Cölbe, Germany). Cells were used for the subsequent experiments at passage 3 as described [22], [23]. Briefly, 200.000 cells/well were seeded to confluence in a 24-well plate (Greiner, Frickenhausen, Germany). After 24 h the medium was replaced with fresh medium containing 1 mM L-ascorbic acid 2-phosphate (Sigma, Deisenhofen, Germany) and the indicated additives. Additives like skin fibroblast decorin (5 µg/ml) and decorin core (Life Cell) were added to the medium. Cells were cultured for 3, 6 or 14 days and medium containing the supplements and decorin or decorin core was changed every other day.

### 2D Gel Electrophoresis and Peptide Mass Fingerprinting

Protein lysates were harvested from 12 well Dcn^−/−^ cultures at 3 day using rehydration buffer. The isoelectric focusing was carried out with ZOOM IPGRunner Kit (Invitrogen) according to the manufacturer’s instructions. Zoom Stripes with a pH 3–10 range were used overnight followed by SDS-gel electrophoresis [43]. After colloidal Coomassie [44] staining all 3 samples were evaluated. Spots were sent to the Centre for Molecular Medicine Central Bioanalytics, Cologne, and analyzed by peptide mass fingerprinting.

### Immune Fluorescence Detection of the Intracellular Filament System and Decorin

For immune fluorescence analysis cells were seeded in µ-Slide VI (ibidi, Martinsried, Germany). All the following procedures were carried out directly in the µ-Slides. At day 6, cells were fixed with 0.5% paraformaldehyde/PBS for 10 min and permeabilized with 1% Triton X-100 in PBS for 5 min. Nonspecific binding sites were blocked with 3% BSA/PBS for 20 min. Immune fluorescence staining was performed for collagen type I, decorin, vimentin, P-Ser72 vimentin, F-actin and Ki67 1:100 in 1% BSA/PBS at 4°C overnight. After washing with PBS the primary antibodies were detected with Alexa 488 goat anti-rabbit, Alexa 488 goat anti-mouse or Cy3 donkey anti-rat 1∶1000 in 1% BSA/PBS containing 1 µg/ml DAPI (Sigma, Deisenhofen, Germany) for 1 h at room temperature. Fluorescence was monitored by a Zeiss Axiovert 200 M microscope (Carl Zeiss AG, Jena, Germany). 3D imaging through the complete matrix was done with an OptiGrid Structured Light-System (Qioptiq LINOS Inc, New York) and Volocity (Improvision/PerkinElmer, Waltham, Massachusetts).

### Quantification of Fluorescence Signals

To quantify the fluorescence signals in the 3D cultures control slides were analyzed at different exposure times [45]. All images were taken at the same exposure time with gain settings in the non saturated range. Digital images of control, decorin treated and decorin core-treated cells (for each condition n≥10 per experiment) were acquired with a Zeiss Axiovert 200 M microscope (Carl Zeiss AG, Jena, Germany) [6]. 3D imaging through the complete matrix was achieved with an OptiGrid Structured Light-System (Qioptiq LINOS Inc, New York) and monitored with Volocity (Improvision/PerkinElmer, Waltham, Massachusetts). Coloured Z-axis layers were merged and re-converted to 8-bit grayscale images without any further digital processing. Values were calculated using ImageQuant (Molecular Dynamics). The obtained volumes were normalized to the cell number of each image indicated by DAPI staining of nuclei.

### Production and Purification of Human Decorin

Decorin was purified under native conditions from conditioned medium of human skin fibroblasts as described before [23], [46]. Briefly, human decorin was purified via the glycosaminoglycan chain using a weak ion-exchanger. 1 L conditioned media was concentrated using a DEAE-Ceramid column (Pall Life Science, Dreieich, Germany) and eluted with 1 M NaCl in 20 mM TRIS buffer followed by HPLC-DEAE eluted with a discontinuous NaCl gradient. The amount of purified proteoglycan decorin from 1 L conditioned medium was 1–1.5 mg, containing about 50–55% dermatan sulphate [16]. The purity of decorin was verified by silver staining after SDS gel electrophoresis. Purification and characterization of the biologically active human decorin protein-core was described before [47]. Briefly, recombinant decorin protein core was purified from conditioned media of a stably transfected 293HEK EBNA cells via the N-terminal His x 6 Tag using a nickel-chelating column. The proteoglycan decorin and the decorin core were subsequently separated by anion-exchange chromatography. Decorin, decorin core and different GAG preparations were analysed for endotoxins with E-TOXATE™ kit according to the manufactures instructions (Sigma, Germany). Minute quantities of endotoxins can cause the E-TOXATE reagent to gel. We analyzed the samples at a 4 times higher concentration than that used in the experiments. Decorin, decorin core and the GAGs did not induce a gel formation indicating that the samples contain no endotoxin or at concentration below the limit of detection (0.05 endotoxin units).

### Atomic Force Microscopy

Contact mode AFM on cells was performed as described before [48]. In this study, cells were chemically stabilized by glutardialdehyde fixation (1% final concentration). Briefly, AFM measurements were carried out in PBS (pH 7.4) using a MultiMode AFM with Nanoscope III controller and software version 5.30sr3 (Digital Instruments, Santa Barbara, CA, USA). Silicon-nitride tips on V-shaped gold-coated cantilevers were used (0.01 N/m, MLCT, VEECO, Mannheim, Germany). Imaging of fixed cells was performed at ambient temperature with forces around 1 nN at 1–3 scan lines per second (1–3 Hz) and 512*512 pixels resolution. Images were processed using the software SPIP, version 3.3.9 by S. Jorgensen (Lyngby, Denmark).

### mRNA Extraction and Quantitative Real-time PCR

For gene expression analysis cells were seeded in 24-well plates (Greiner, Frickenhausen, Germany). Total RNA was extracted from cells at day 3, 6 and 14 using RNeasy Mini Kit (Qiagen, Hilden, Germany). cDNA was synthesized from 2 µg of total RNA using Omniscript RT Kit (Qiagen). mRNA levels of target genes were quantified by Real-Time RT-PCR using an ABI PRISM 7500 Sequence Detector (Applied Biosystems) (core unit IZKF Münster) and MESA GREEN (Eurogentec, Köln, Germany). Amplification was performed in triplicate in a total volume of 25 µl. Raw data were normalized on geometric average of control genes [49]. The primer pairs were designed to overlap intron/exon borders and to minimize primer-dimers. Annealing temperature was calculated to be identical for all primer pairs. Nucleotide sequence of the primers used in this study: vimentin-F 5′-CTGTGTCCTCTGCCTCCTA-3′; vimentin-R 5′-CCACTTTCCGTTCAAGGTCAAG-3′; integrin beta 1-F 5′-CAA GAG GGC TGA AGA TTA CC-3′; integrin beta 1-R 5′-GGC ATC ACA GTT TTA TCC A-3′; actin-F 5′-GGGTGTGATGGTGGGAATGG-3′; actin-R 5′-TGGCTGGGGTGTTGAAGGTC-3′; GAPDH-F 5′-ATTCAACGGCACAGTCAAG-3′; GAPDH-R 5′-TTCACACCCATCACAAACAT-3′; ubiquitin c-F 5′-GCCCAGTGTTACCACCAAG-3′; ubiquitin c-R 5′-CACCAAAGAACAAGCACAAG-3′. F-forward primer, R-reverse primer.

### Protein Extraction and Western Blotting

For protein analysis cells were seeded in 12-well plates (Greiner, Frickenhausen, Germany). At day 3, 6 and 14 cells were lysed with 200 µl/well urea buffer (40 mM Tris, 7 M urea, 2 M thiourea and 1% ASB-14 (Sigma, Deisenhofen, Germany)) for 10 min on ice. Total protein lysates were dissolved in SDS sample buffer, separated on 12% gels and transferred to PVDF membranes. After blocking with 5% milk powder/TBS-Tween for 1 h the membranes were incubated with antibodies against the target proteins fibronectin, β1 integrin, α2 integrin, α5 integrin, vimentin, P-Tyr/Ser/Thr and GAPDH 1∶1000 in 2.5% milk-powder/PBS at 4°C over night. After washing with TBS-Tween the membranes were incubated with the secondary antibody (anti-rabbit-HRP, Amersham; anti-goat-HRP, Millipore) for 1 h. Proteins were visualized using ECL Western blotting detection reagents (Pierce, Rockford, USA) and Fusion-SL 4.2 MP (PeqLab, Erlangen, Germany). Grey-scale values were calculated using ImageQuant (Molecular Dynamics) and target proteins were related to GAPDH or total protein amount visualized by SDS-PAGE and Coomassie staining.

### Metabolic Labeling and Immune Precipitation

Dcn^−/−^ cells were seeded in 6-well plates and cultured for 6 days as described above. Cells were washed with PBS and 1 ml/well cysteine/methionine-free MEM (GIBCO) containing 10% FCS was added. After addition of 100 µCi/ml L-^35^S-Cysteine and L-^35^S-Methionine cells were incubated over night at 37°C. Matrices were washed trice with PBS and harvested in 500 µl/well lysis buffer (150 mM NaCl, 25 mM Tris, 5 mM EDTA, 0.5% NP_40_, proteinase-inhibitor, pH 7.4). Cell disruption was done at −20°C. Lysates were centrifuged (5 min, max rpm, 4°C) and pre-cleared each with 10 µl rabbit serum for 30 min at 4°C. Metabolic labeling was verified by determination of [^35^S] incooperation under all three conditions. Protein A sepharose was equilibrated with buffer (20 mM NaH_2_PO_4_, 150 mM NaCl, proteinase-inhibitor, pH 8.0) and 10 µl of this suspension added to each lysate. After incubation at 4°C for 30 min, lysates were centrifuged for 1 min at 10.000 rpm. Vimentin antibody (BioVision) was added to the supernatant in a 1∶10 dilution and incubated overnight at 4°C. Equilibrated protein A sepharose was blocked with 2% BSA/PBS and then 50 µl were added to each lysate. After incubation at 4°C for 2 h precipitates were centrifuged for 1 min at 10.000 rpm. The immune complex was washed thrice with buffer I (400 mM NaCl, 25 mM Tris, 5 mM EDTA, 0.5% NP40, proteinase-inhibitor) and one time with buffer II (125 mM Tris, proteinase-inhibitor, pH 6.8). The immune precipitation of vimentin was confirmed by determination of [^35^S]. 40 µl SDS sample buffer (5×, reduced) were added and samples were separated in a SDS-PAGE (4.5–15%) and fixed for 10 min in 25% methanol/7% acetic acid. After incubation in 1 M sodium-salecylate for 1 h the gel was washed thrice with dH_2_O and dehydrated in 20% ethanol/3% glycerine overnight. The gel was dried and the fluorographs were developed at −80°C.

### Measuring pH at the Cell Surface

The cell surface pH of Dcn^−/−^ cells was measured as previously described for B16 melanoma cells [50]. Initially, cells were seeded on collagen I and cultured for 24 h. The glycocalix was then labeled with 12.5 µg/ml wheat germ agglutinin (WGA, Molecular Probes) in HEPES-buffered Ringer solution [51]. pH was measured ratiometrically using the Metafluor software (Visitron Systems GmbH, Puchheim, Germany). Cells were continuously superfused with prewarmed (37°C) Hepes-buffered Ringer solutions of pH 7.2. The excitation wavelength for WGA alternated between 440 nm and 490 nm while the emitted fluorescence intensities were monitored at 510 nm using a Photometrics camera (CoolSnap*fx*, Visitron Systems). A ratio was calculated from the intensities measured at 440 and 490 nm. Thus, the measurement was virtually independent of the amount of dye excited in a given region of interest and represented the local proton concentration. Fluorescence intensities were measured in 35 second intervals and corrected for background fluorescence by subtracting background intensities of control regions placed right next to the measured regions of interest. At the end of each experiment, the pH measurements were calibrated applying the nigericin/high [K^+^] method as published [50].

### Blocking of ß1 Integrin

To analyse the effect of blocking the β1 integrin subunit, cells were incubated with Low Endotoxin Azide Free (LEAF™) β1 integrin blocking antibody (5 µg/ml) for 6 h at 37°C. Cells were washed twice with PBS and harvested with urea containing lysis buffer. Vimentin expression was analysed by Western blotting as described above.

## Results

### The Loss of Decorin Affects Vimentin Expression in vitro and in vivo

**Figure 1:**
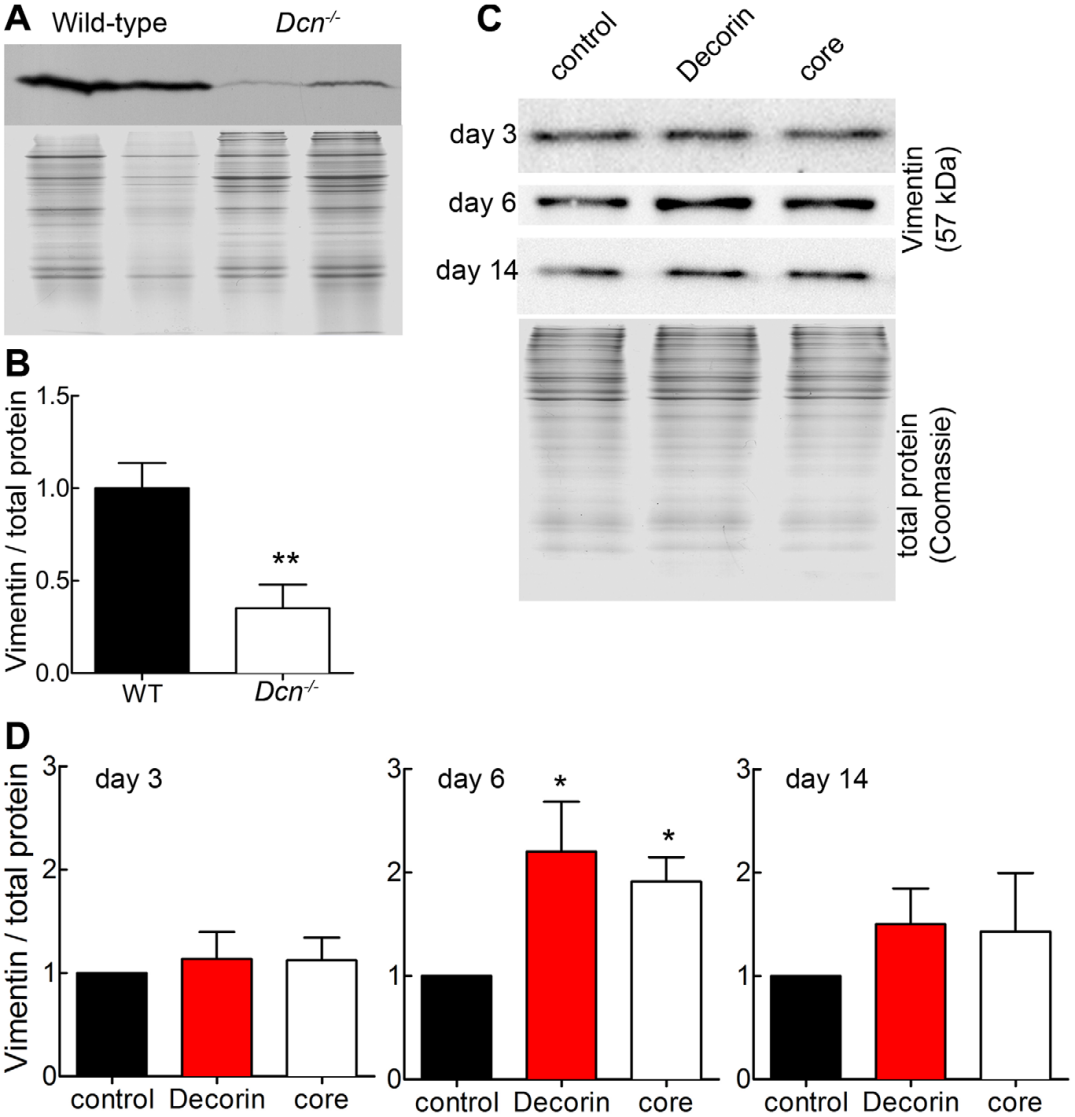
Detection of vimentin *in vivo* in Dcn^−/−^ and wild-type mouse skin and *in vitro*. (A) Western blot for vimentin (57 kDa) with two different skin extracts of adult wild-type and Dcn^−/−^ mice (upper panel). Coomassie gel was used as loading control and shows the corresponding protein extracts (lower panel). (B) Quantification of Western blots, where the vimentin signal was normalized to the Coomassie gel staining (n = 4; **, p<0.01). (C) Dcn^−/−^ fibroblasts were cultured for 3, 6 and 14 days in the presence of ascorbate-2-phosphate and treated with decorin or decorin core (core) and the respective controls. Protein extracts were immunoblotted with antibodies to detect vimentin. Coomassie gel was used as loading control (lower panel, exemplary). (D) Quantification of vimentin protein expression at day 3, 6 and 14. Western blot signals were normalized to Coomassie staining. Data represent 3 independent experiments and are expressed as mean ± SD (*, p<0.05).

To investigate the effects of decorin and dermatan sulfate on cell function, we used an in-vivo-like cell culture system consisting of Dcn^−/−^ dermal fibroblasts synthesising their own collagen-rich matrix and supplemented with decorin or decorin protein core. Two-D protein screening followed by peptide mass fingerprinting of Dcn^−/−^ fibroblasts treated with decorin showed that vimentin was regulated differentially when compared to non-treated cells (Figure S1). Immunofluorescence (data not shown) and Western blot analysis of dermal extracts of adult Dcn^−/−^ and wild-type mice showed that vimentin was significantly reduced by the ablation of decorin (Figure 1A, B). We confirmed by qRT-PCR that wild-type fibroblasts express significantly more vimentin than Dcn^−/−^ fibroblasts when the cells are 3 days in culture and therefore in a 2D environment (Figure S2). Surprisingly, treating Dcn−/− fibroblasts with decorin at this time point results in a significant reduction of vimentin mRNA, supporting the idea, those fibroblasts should be in a 3D environment (Figure S2).

To evaluate the screening and the in vivo results at a molecular level, we analyzed matrix formation in cultures of Dcn^−/−^ fibroblast. Dcn^−/−^ fibroblasts were treated with either the proteoglycan decorin or its core in the presence of ascorbate-2-phosphate to generate altered collagen-rich matrices. To study the influence of the GAG chain, the decorin protein core was used. At day 3, 6 and 14, the Dcn^−/−^ fibroblasts inside their own matrix were analysed by Western blotting for vimentin expression (Figure 1C). We have previously shown that at day 6, the cells already produce matrix molecules like collagen I, III and V and deposit these molecules around the cells [22]. Here we found a significant increase in vimentin caused by decorin treatment (Figure 1D). Therefore, exogenous decorin can rescue the vimentin IF system in the Dcn^−/−^ cultures. Interestingly, treatment with the decorin core revealed also a significant increase of vimentin but less effective as the decorin treatment.

**Figure 2:**
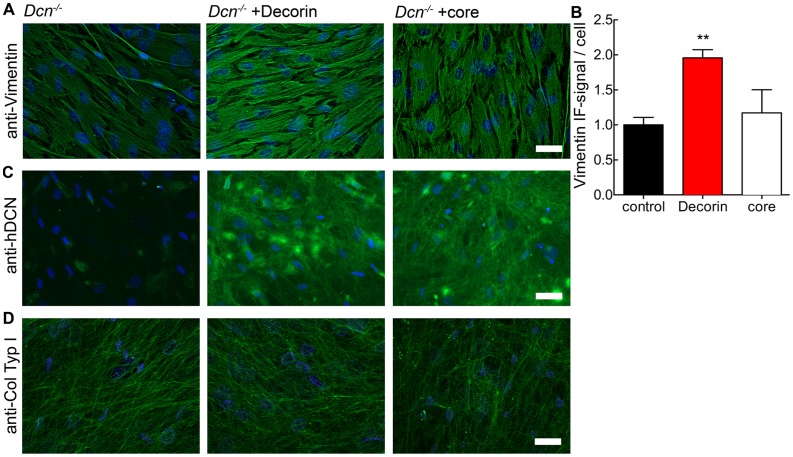
Expression of vimentin in 3D cultures of Dcn^−/−^ fibroblasts treated with either decorin or decorin core (core) at day 6. Immunofluorescence staining of vimentin (A), decorin (C) and collagen type I (D) in µ-slide VI, visualized by Alexa 488 conjugated secondary antibody (green). Nuclei were stained with DAPI (blue). Images show merged z-axis layers of the complete 3D matrix. B: Quantification of vimentin immunofluorescence signal in merged layers normalized to cell number (nuclei) per image. Data represent 5 independent experiments and are expressed as mean ± SD (for each condition and experiments, 15 images were evaluated; **, p<0.01). Bar = 100 µm.

Next, we visualised the vimentin IF system using immunofluorescence microscopy on cells cultured on µ-slides (Figure 2A). Quantification of the fluorescence signal showed that added decorin increased the amount of vimentin in the Dcn^−/−^ fibroblasts (Figure 2B), while the effects of the decorin protein core were less dramatic. The analysis of (i) the actin expression by qRT-PCR and (ii) the actin cytoskeleton by phalloidin staining or atomic force microscopy showed no alterations in decorin treated or untreated fibroblasts (Figure S3). Also when cultured in µ-slides Dcn^−/−^ fibroblasts produce their own collagen-rich matrix (Figure 2D) and incorporate the proteoglycan decorin as well as the decorin protein core (Figure 2C). Decorin synthesis by wild-type fibroblasts and deposition into the matrix was clearly detectable after 4–6 day (data not shown). Of note is the altered organization of vimentin in Dcn^−/−^ fibroblasts compared to cultures treated with decorin and its core.

### Decorin-deficiency Influences the Nuclear Morphology in Dcn^−/−^ Cells

**Figure 3:**
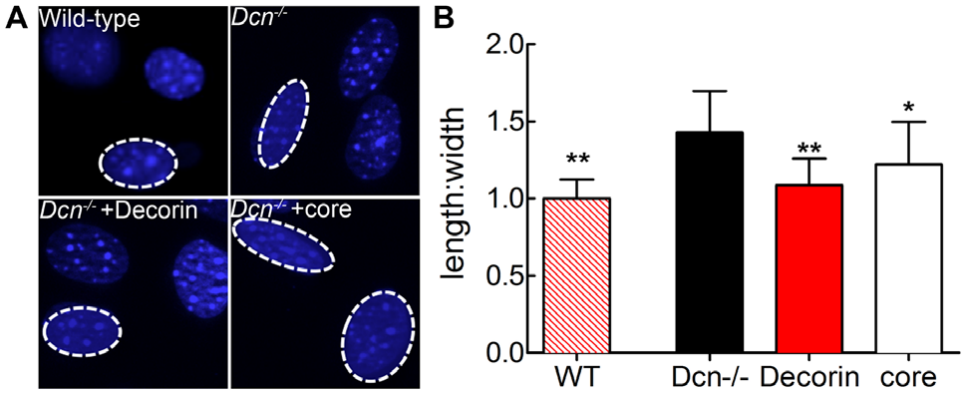
Analysis of nuclear morphology index. (A) Nuclei in 3D cultures of Dcn^−/−^ fibroblasts treated with either decorin or decorin core (core) as well as 3D cultures of wild-type fibroblasts were stained with DAPI (blue) at day 6 in µ-slide VI. (B) Length and width of nuclei were measured using measureIT 5.1 (Olympus) and the length:width ratio was determined. The value of wild-type nuclei was set at 1 and corresponds to a more circular shape of the nuclei. Student’s *t* test (unpaired) revealed significant differences between the Dcn^−/−^ fibroblasts compared to wild-type and the decorin or decorin core treated samples. Data represent 3 independent experiments and are expressed as mean ± SD (for each condition, a total of 50 nuclei were measured; *, p<0.05; **, p<0.01).

In preliminary experiments we noticed that upon decorin treatment, the nuclei of the Dcn^−/−^ fibroblasts actively producing matrix were quite elongated as compared to controls. To investigate this in more depth, we first cultured fibroblasts from various genotypes for 6 days, labeled the nuclei with the fluorescence dye DAPI and analyzed the cells by fluorescence microscopy. 3D imaging through the complete matrix was done with an OptiGrid Structured Light-System (Qioptiq LINOS Inc, New York). Colored Z-axis layers were merged. Interestingly, the nuclear morphology was different in the decorin and decorin core treated 3D cultures as compared to untreated Dcn^−/−^ cells. Wild-type fibroblasts displayed a round morphology different to the ellipsoid form of the Dcn−/− fibroblasts (Figure 3A). To quantify these differences in the nuclear morphology, we determined the morphology index [52] as a ratio of length to width for wild-type and Dcn^−/−^ fibroblasts (Figure 3B). The wild-type form resulted in a morphology index of 1 significantly different from the 1.5 of Dcn^−/−^ fibroblasts. Exogenous addition of decorin leads to significant changes in the index to 1, which is similar to the wild-type. The treatment with decorin protein core also significantly changed the index but not as prominent as decorin (Figure 3B). It has been shown for fibroblasts that the nuclear shape can be correlated with overall cell shape [52]. Decorin treatment resulted in a complete rescue of the morphology index, whereas the decorin core only partially rescued the phenotype. This suggests that the extracellular dermatan sulfate proteoglycan decorin has a different effect on the cells than the decorin protein core.

### Only the Proteoglycan Decorin Treatment can Modulate α2β1 Integrin Expression in Matrix-producing Dcn^−/−^ Fibroblasts

**Figure 4:**
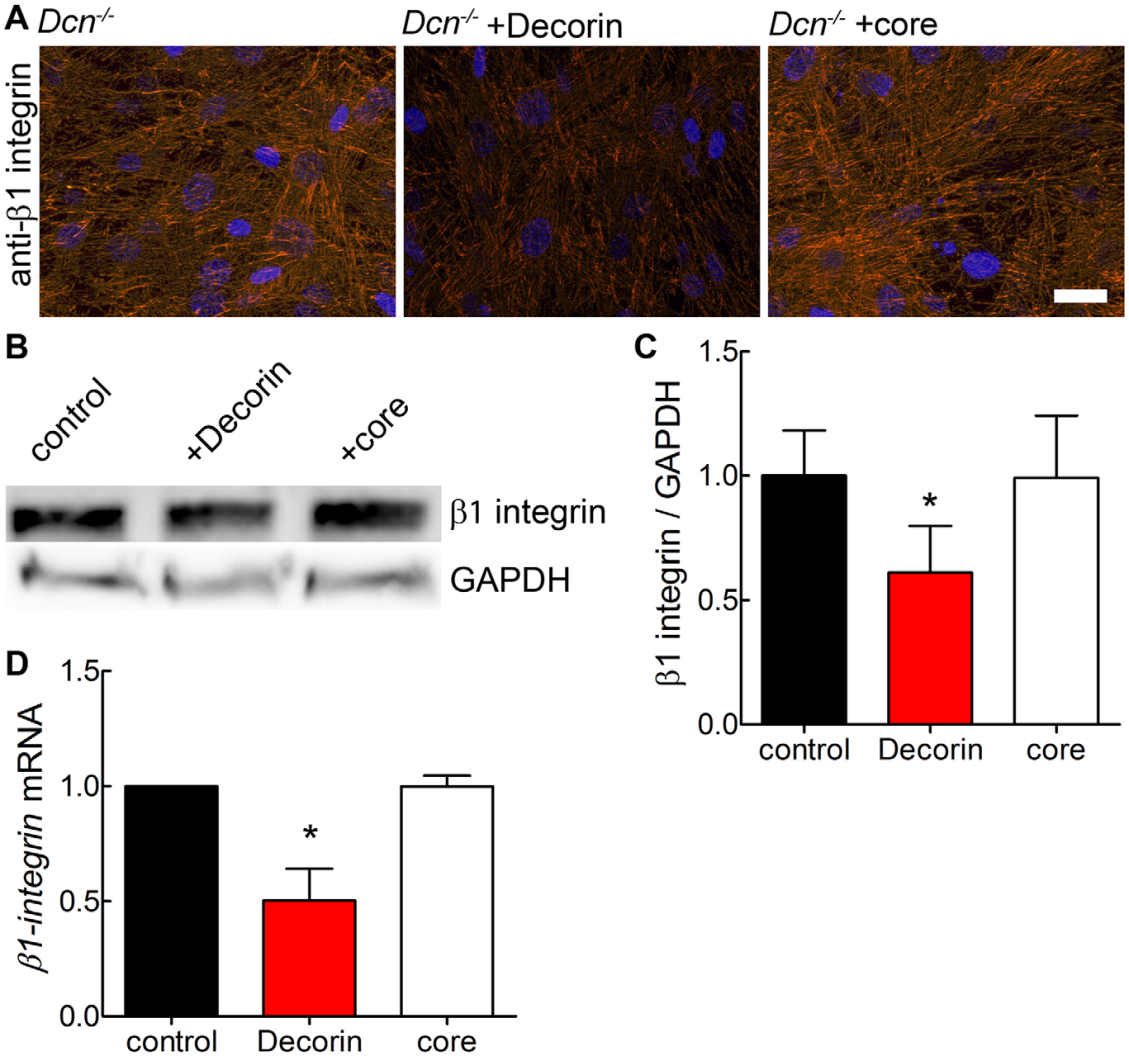
Modulation of β1 integrin in 3D cultures of Dcn^−/−^ fibroblasts at day 6 by the DS proteoglycan decorin. (A) Immunfluorescence staining of Dcn^−/−^ fibroblasts for β1 integrin in µ-slide VI after treatment with either decorin or decorin core (core), visualized by Cy3 conjugated secondary antibody (red). Nuclei were stained with DAPI (blue). Images show merged z-axis layers of the complete 3D matrix (bar = 100 µm). (B) Representative Western blot of 3D cultures extracts for β1 integrin and the respective control GAPDH (lower panel). (C) Quantification of β1 integrin signals shown in B in grey-scale values of Western blot signals and normalized to GAPDH as loading control. Student’s *t* test (unpaired) revealed a significant difference for decorin treated cells compared to controls. Data represent 3 independent experiments and are expressed as mean ± SD (*, p<0.05). (D) Quantification of β1 integrin mRNA-levels by qRT-PCR. CT values were normalized to reference genes as described in Materials & Methods. Data represent 3 independent experiments and are expressed as mean ± SD (*, p<0.05).

Decorin in the soluble form is known to bind to several cell surface receptors [53]. Decorin can modulate α2β1 integrin activity which requires the presence of the GAG chain [26], [27]. α2β1 integrin is a collagen receptor and links the ECM to the cytoskeleton [24], [54]. To test whether decorin would affect β1 integrin expression, we used immunofluorescence and Western blot analysis. Decorin was partially co-localized with β1 integrin in the 3D Dcn^−/−^ cultures treated with decorin (Figure S4A) and similarly in the 3D wild-type fibroblast cultures (Figure S4B). Exposure to the decorin proteoglycan caused a marked suppression of β1 integrin immunoreactivity in Dcn^−/−^ fibroblast cultures (Figure 4A,B). In contrast to the proteoglycan, the decorin core had no effect (Figure 4B,C). Interestingly, also the decorin treatment led to a significant reduction of β1 integrin in wild-type fibroblasts after 6 days in culture (Figure S4C,D), however to a lesser extent. We could also confirm, that the lack of decorin in the dermis of Dcn^−/−^ mice leads to significantly more β1 integrin protein compared to wild-type (Figure S5). For the β1 integrin we could show that in Dcn^−/−^ fibroblasts decorin treatment reduced mRNA expression (Figure 4D), which could be an explanation for the reduction of the β1 integrin protein.

**Figure 5:**
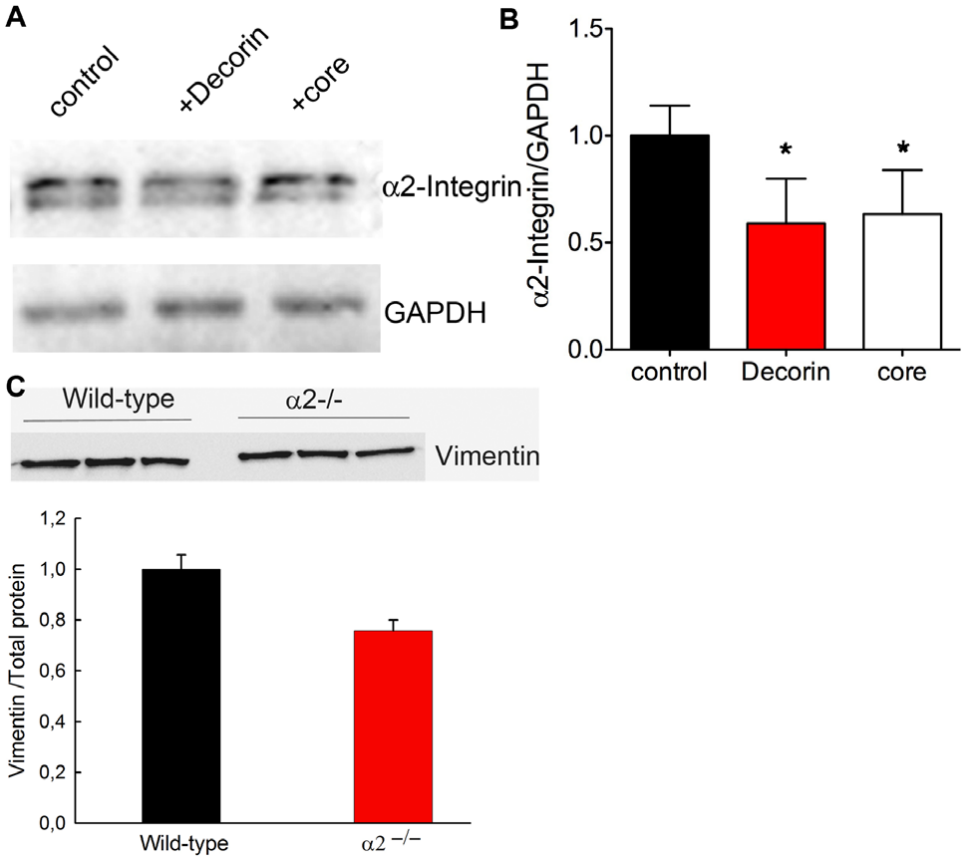
Influence of decorin on the a2 integrin subunit. (A) α2 integrin subunit expression in Dcn^−/−^ fibroblasts cultured for 6 days in the presence of decorin or decorin core and the GAPDH control (lower panel). (B) Quantification of α2 integrin Western blot signals normalised to GAPDH (3 independent experiments; *, p<0.05). (C) Analysis of α2^−/−^ fibroblasts compared to wild-type by Western blot and the quantification of vimentin normalised to total protein.

It is known that decorin as a proteoglycan can modulate α2β1 integrin, but not α1β1 activity [27], therefore, we examined the expression of α2 subunits in Dcn^−/−^ fibroblasts. Decorin treatment led to a significant reduction of α2 integrin indicating that decorin affects the amount of α2β1 integrin in the Dcn^−/−^ cultures (Figure 5A,B). Interestingly, the decorin core altered also the expression of the α2 integrin, which is in contrast to the β1 expression. Another β1 integrin partner is the α5 subunit. No changes in α5 integrin and fibronectin, the respective ligand were detected in Dcn^−/−^3D fibroblasts cultures (Figure S6).

To determine whether the loss of decorin may influence the amount of vimentin, via α2β1 integrin, fibroblasts deficient in α2β1 [41] were cultured for 6 days in the presence of ascorbate-2-phosphate followed by Western blot for vimentin. The quantification and normalization to total protein amounts showed a ∼30% reduction of vimentin in integrin α2-deficient fibroblasts compared to the controls (Figure 5C). The reduction of vimentin was confirmed in α2β1-null skin compared to wild-type skin by immunofluorescence (data not shown).

### Only the DS Proteoglycan Decorin, but not the Decorin Core Increases the Pericellular H^+^ Concentration

**Figure 6:**
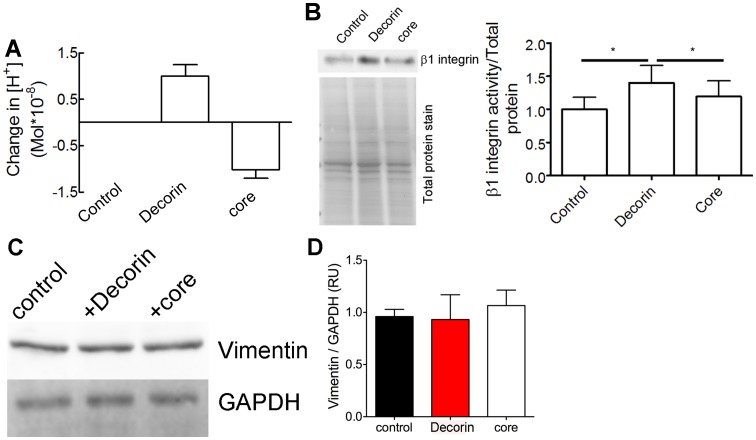
Cell surface proton concentration and activity of β1 integrin in Dcn^−/−^ fibroblasts is altered by decorin treatment. (A) Determination of the proton concentration on the cell surface after treatment with 0.3125 µg/ml decorin and decorin core (core). Dcn*^−/−^* fibroblasts were cultured over night on collagen type I coated wells as described in Materials and Methods (Decorin n = 22 cells; core n = 33 cells; *, p<0.05, n.s. = not significant). (B) Western blot with β1 integrin antibody 9EG7 that recognizes the active form of β1 integrin of Dcn^−/−^ fibroblasts cultured for 6 days in the presence of ascorbate-2-phosphate and decorin or decorin core (core) (upper panel). The quantification of the Western blot for active β1 integrin is normalised to total protein stain (lower blot) (3 independent experiments; *, p<0.05, paired t’test). (C) Western blot for vimentin of Dcn^−/−^ fibroblasts cultured for 6 days in the presence of ascorbate-2-phosphate and decorin or decorin core (core). 6 h prior to harvesting the cells were treated with a β1 integrin blocking antibody and analysed for vimentin (upper panel). The loading control GAPDH is shown in the lower panel. (D) Quantification of the Western blot for vimentin normalised to GAPDH. Data represent 3 independent experiments and are expressed as mean ± SD.

Since decorin and decorin core had distinctly different effects on β1 integrin we focused on the response of fibroblasts to post-translational modifications of decorin. We showed previously that DS decorin reduces the pericellular pH of melanoma cells [50]. In addition to structure and constituents of the ECM, also the pH is considered to have an impact on integrin activation [55]. Therefore, we measured the pericellular proton concentration in Dcn^−/−^ fibroblasts seeded on collagen I. Treatment with decorin and decorin core revealed different effects on the cell surface proton concentration. DS decorin led to a significant increase in the proton concentration, which could be due to the DS chain whereas the decorin core resulted in an alkalinisation (Figure 6A). The difference in the protein core of DS decorin and decorin core is a His x6-Tag at the N-terminus of the decorin core which can be protonated. These results suggest a different, pH-mediated impact of the decorin proteoglycans and decorin core on matrix receptors such as β1 integrins, indicates that the different forms of matrix molecules might exert distinct and specific modulatory effects. To demonstrate that DS decorin and the core differentially modulate β1 integrin levels, we determined levels of active β1 integrin using a specific antibody (clone 9EG7) that recognizes the active conformation [56] in 6 day cultures of Dcn^−/−^ fibroblasts. The Western blot for β1 integrin activity showed a clear difference depending on the source of decorin (Figure 6B left panel). As expected the core protein had no effect on levels of active β1 integrin, while DS decorin clearly increased active β1 integrin (Figure 6B, right panel).

To demonstrate a mechanistic link between decorin, β1 integrin and the vimentin IF in our 3D Dcn^−/−^ fibroblasts, cells were cultured as described above, but in the presence of a β1 integrin function-blocking antibody for the last 6 h [57]. In vitro it has been shown that the phosphorylation required for assembly and disassembly of vimentin is a fast and dynamic process leading to changes in the IF system within 80 min [58]. Western blot analysis for vimentin showed that decorin and decorin core had no effect on the vimentin expression levels after blocking β1 integrin (Figure 6C,D). Thus, we conclude that in Dcn^−/−^ fibroblasts, addition of decorin stabilizes the vimentin IF and this, in turn, might also affect α2β1 integrin expression during the synthesis of a collagen-rich matrix (Figure 4D). Together we can show that depending on the presence of the GAG chain the effects of the decorin on α2β1 integrin levels and activity is different supporting the importance of the proteoglycan decorin.

### Vimentin mRNA Expression and Phosphorylation is Increased by Decorin Core Treatment but not by Decorin

**Figure 7:**
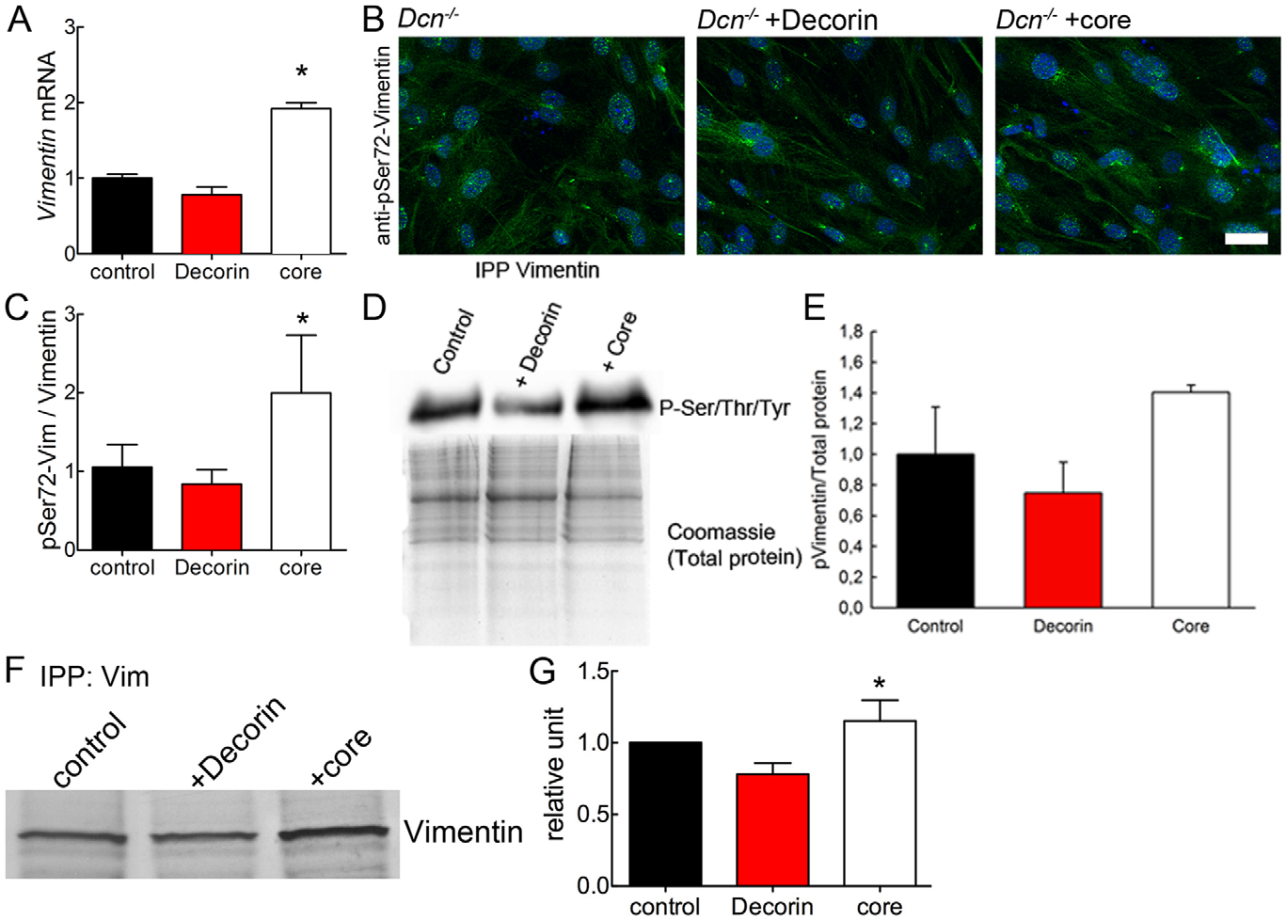
Analysis of vimentin synthesis and degradation in 3D cultures of Dcn^−/−^ fibroblasts at day 6 treated with either decorin or decorin core (core). (A) Quantification of vimentin mRNA expression with qRT-PCR (n = 3 independent experiments; *, p<0.05). CT values were normalized to reference genes. (B) Immuno fluorescence staining for phospho-vimentin P-Ser72 in µ-slide VI visualized by Alexa 488 conjugated secondary antibody (green). Nuclei were stained with DAPI (blue). Images show merged of z-axis layers of the complete 3D matrix (bar = 100 µm). (C) Quantification of phospho-vimentin immunofluorescence signal in merged layers normalized to cell number (nuclei) per image and related to total amount of vimentin (Fig. 5) (n = 3 independent experiments; for each condition, 15 images were measured). Student’s *t* test (unpaired) revealed differences between the core-treated and the control cells. Data are expressed as mean ± SD (n = 3 independent experiments; *, p<0.05). (D) Vimentin immunoprecipitation followed by a Western blot for total sulfation (P-Ser/Thr/Tyr). Coomassie gel for total protein is shown in the lower panel. (E) Quantification of P-vimentin normalized to total protein amounts. Data represent 3 independent experiments and are expressed as mean ± SD. (F) Dcn^−/−^3D cultures were metabolically labeled for 12 h with 100 µCi/ml ^35^S-Methionine/^35^S-Cysteine followed by fluorography. Immunoprecipitation with vimentin antibody showed the de novo synthesis is increased by decorin protein core treatment. (G) Quantification of vimentin de novo synthesis. Data are expressed as mean ± SD (n = 3 independent experiments; *, p<0.05).

Treatment of Dcn^−/−^ fibroblasts with decorin or decorin core for 6 days revealed that only decorin core was capable of significantly increasing the vimentin mRNA levels (Figure 7A). This result demonstrates once more that the molecular role of the proteoglycan decorin is different from that of the decorin core. The proteoglycan had no effect on the expression of vimentin mRNA even though the amount of protein was significantly increased at day 6 (cfr. Figure 1D) probably due to an accumulation in decorin treated Dcn^−/−^ cultures. Surprisingly, at day 3 in culture the proteoglycan decorin-treatment led to a reduced amount of vimentin mRNA, indicating that the matrix synthesised by the cells is affecting the function (Figure S2).

The phosphorylation of vimentin is necessary for the assembly and disassembly of the existing filament system [58]. The phosphorylation occurs at specific sites in the head-domain, for example at Ser72. The Dcn^−/−^ fibroblasts were cultured in µ-ibidi slides for 6 days and stained for P-Ser72 and vimentin (Figure 7B). The fluorescence signal was determined for P-Ser72 as well as for vimentin and the quantification was normalised to the amount of vimentin (Figure 7C). Interestingly, the amount of P-Ser72 was increased in decorin core-treated cultures and not in Dcn^−/−^ fibroblasts.

To this end, Dcn^−/−^ fibroblasts were metabolically labelled for 12 h with a mixture of ^35^S-Methionine and ^35^S-Cysteine. The incorporation of radioactive tracer was similar in decorin and decorin core treated cultures. Afterwards, the cells were subjected to immune precipitation at 4°C overnight followed by SDS-PAGE and radio fluorography. The vimentin de novo synthesis was increased by the decorin core compared to decorin-treated and control samples (Figure 7F,G). These findings are supported by the increase in mRNA and further corroborate our hypothesis that only the proteoglycan decorin can stabilize the vimentin IF, thereby supporting tissue integrity. In addition, a matrix lacking the GAG chain of decorin leads to an increased turn-over of vimentin and might therefore not be able to completely stabilize the IF.

## Discussion

In the present study, we provide evidence that the DS proteoglycan decorin can modulate α2β1 integrin during matrix synthesis and can influence cell morphology via stabilizing the vimentin intermediate filament system. In vivo the loss of decorin in the dermis leads to a reduced amount of vimentin and to an increase in β1 integrin. The link between α2β1 and vimentin was confirmed by reduced vimentin levels in fibroblasts lacking α2β1 integrin. These findings could provide a reasonable explanation for the delayed wound healing and atrophic scaring seen in Dcn^−/−^ mice, the reduced tissue integrity and the EDS phenotype.

The dermis of Dcn^−/−^ mice is thinner, shows an altered organization of fibrillar collagens and a delay in wound healing [20], [21]. To study the influence of decorin or its DS chain on cell behavior we used a 3D cell culture model where Dcn^−/−^ fibroblasts produce their own collagen-rich matrix [22]. This is a suitable model that resembles the in vivo situation [25]. These cultures indeed represent the in vivo fibrillar collagen phenotype of the Dcn^−/−^ mice [20] that can be rescued by addition of decorin [22], [23]. Interestingly, by addition or viral expression of the decorin phenotype of the fibrillar collagens in cell culture is similar to the dermatan C5-epimerase1^−/−^ mice [19]. By comparison of 2D gels of Dcn^−/−^ fibroblasts treated with decorin or decorin core we detected changes in components of the intracellular vimentin intermediate filament system.

Evaluation of the differentially expressed IF protein vimentin showed that Dcn^−/−^ fibroblasts in vitro contain less vimentin as compared to decorin-treated fibroblasts. This could be confirmed in vivo as wild-type dermis contained more vimentin compared to Dcn^−/−^ skin. Vim^−/−^ mice develop and behave normally [38]. However, these mutant mice display also delayed wound healing [40] similarly to the Dcn^−/−^ mice [21]. Vimentin is required for cell movement depending on the generation of significant traction forces. The delay in wound healing of Vim^−/−^ mice could be correlated with reduced tissue integrity and reduced migration of the Vim^−/−^ fibroblasts [39]. Whether this also occurs in Dcn^−/−^ fibroblasts is difficult to assess, because, in our system the fibroblasts do not migrate but instead they synthesis matrix. Therefore, these similarities suggest that a decorin-containing ECM stabilizes the vimentin IF system only if the DS chain is present. Accordingly, EDS patients with partial lack of decorin DS chain display altered wound healing with atrophic scarring [15]. In contrast to the DS proteoglycan decorin, the decorin protein core only partially rescued the vimentin IF system. IF turnover occurs along the length of the filaments, unlike that of microfilaments or microtubules. Usually, there is only little IF protein found in the soluble fraction [29]. We used µ-ibidi slides to avoid the problem of solubility and supported the results obtained by immuno blotting that decorin evokes vimentin deposition. By detecting a significant increase in vimentin mRNA, phosphorylation and de novo synthesis we demonstrate that the lack of the DS chain of decorin increases the vimentin IF turnover during matrix synthesis. In contrast to Dcn^−/−^ fibroblasts wild-type cells synthesize significantly more vimentin mRNA (Figure S2). Hence, the proteoglycan decorin stabilizes the vimentin IF. Furthermore, the metabolic activity of Dcn^−/−^ cultures was increased upon treatment with decorin or decorin core (data not shown). This could be explained by increased protein synthesis. The decorin core induces production, but is not able to properly deposit vimentin, while decorin increases collagen I production and deposition in the extracellular matrix [22].

Dcn^−/−^ mice show no obvious phenotype during their development likely due to the functional compensation of biglycan and its DS chains [59]. However, after wounding Dcn^−/−^ mice, biglycan is not capable of compensating [21]. Cukiermann and coworkers showed that cells synthesizing their own matrix are closer to the in vivo situation compared to cells seeded in Matrigel or collagen gels [60], [61] or monolayer cultures [25].

Previously, it has been shown that the vimentin IF system contributes to the localization and shape of the nuclei [36] and is involved in the regulation of cell adhesion [32], [33] by binding to the cytoplasmic domain of α2β1 integrin. The loss of decorin results in a more elongated shape of the nuclei, which is in line with increased adhesion of Dcn^−/−^ fibroblasts [4]. Interestingly, addition of decorin normalized the nuclear phenotype of the Dcn^−/−^ cells to the level of wild-type fibroblasts whereas the core protein itself only partially rescued the phenotype. Cell adhesion was shown to regulate the cell shape, which resembles the shape of the nucleus. In a 2D cell system the cell shape of fibroblasts seeded on fibronectin depend on the organization of the actin cap [52]. The fibroblasts used in the present study synthesize a more complex matrix [22], [50] and therefore adhere to a mixture of matrix molecules, which does not lead to changes in the actin structures. Though, Dcn^−/−^ fibroblasts show increased adhesion to collagen I and fibronectin substrates [4].

The link between the collagen-rich matrix and the cell’s interior is provided by integrins containing the β1 subunit [54], [62]. The proteoglycan decorin can modulate α2β1, but not α1β1 integrins on endothelial cells and increase cell adhesion [27]. Also, Dcn^−/−^ dermal fibroblasts express in vivo more β1 integrin compared to control fibroblasts (Figure S3).

The ECM provides the dermis with tensile strength and flexibility. The ECM molecule decorin is not only structural scaffold binding to collagens but also can bind to cell surface receptors or adhesion molecules. Decorin has previously been shown to affect collagen phagocytosis by inhibiting the binding of fibrillar collagen to α2β1 integrin in gingival fibroblasts. In conditions requiring matrix synthesis like in wound healing decorin might thereby help to build up the collagen network [63]. In line with this notion we showed that Dcn^−/−^ fibroblasts synthesize and deposit more collagen I when treated with decorin [22]. In the tissue-forming phase of wound healing, the fibroblasts synthesize collagen and decorin might prevent the phagocytosis of collagen to support matrix synthesis. Our study shows that the addition of exogenous decorin proteoglycan to Dcn^−/−^ fibroblasts during collagen/matrix synthesis and deposition reduces the amount of both the β1 integrin mRNA, possibly due to sequestering TGF- β [64], [14], and α2β1 protein. The reduction of α2β1 integrin by decorin or decorin core could indirectly influence the adhesion of the fibroblasts in their own collagen-rich matrix visible by the nuclear morphology. Nieminen and co-workers showed that the reorganization of vimentin IF in blood cells reflects the role of IF in forming anchoring structures for adhesion molecules on the cell surface [65]. In Dcn^−/−^ mice during DTH, the adhesion of leukocytes to the endothelium is increased but the transmigration is decreased [12]. These results and the interaction of vimentin with the α2β1 cytoplasmic tail [34] support our hypothesis, that the decorin is necessary to stabilize the vimentin IF via α2β1 integrin. Cell adhesion depends on the activity state of integrins, which can assume either of three different activity states: high, low and intermediate affinity conformation [66]. Furthermore, the activity state of integrins depends on the pH. An acidic extracellular pH leads to an increased avidity [55]. In the present study, only proteoglycan decorin treatment leads to a weak acidification of the pericelluar pH. In contrast, the decorin core leads to an alkalinization, and therefore is not influencing β1 integrin activity in a way that would strengthen adhesion. However, DS proteoglycan decorin influenced the activity of β1 integrin in our 3D model and also affects in vitro the activity state of α2β1 integrin [27]. This is in line with previous publications demonstrating that the GAG chain is required for decorin-dependent β1 integrin modulation [26], [27]. Interestingly, the DS proteoglycan decorin inhibits melanoma cell migration by reversibly acidifying the cell surface pH to an intermediate value of 7.1 [50], where migration reaches its maximum [67]. In the present 3D cell culture model that resembles a wound healing model, decorin expression in wild-type fibroblasts is increased at day 4–6 (data not shown). This could alter both the amount and the avidity of β1 integrin and thereby influence cell/matrix interaction in wild-type, but not in Dcn^−/−^ fibroblasts. Decorin from skin fibroblasts contains GAGs of complex, different sulfated DS structures [50]. As phosphorylation of vimentin is increased in decorin core-treated fibroblasts, we hypothesize elevated P-vimentin levels could contribute to a reduced mechanical stability of the matrices. This can be linked to the cutis laxa of EDS patients where decorin is synthesized partially without a GAG chain [15].

In conclusion, we demonstrate for the first time that DS of decorin is required to stabilize the vimentin IF system which is linked to α2β1 integrin. This is in line with the finding that Dcn^−/−^ dermis contains less vimentin compared to wild-type. Furthermore, the lack of decorin leads to an increase in dermal β1 integrin protein. In agreement with these findings, exogenous decorin reduces the amount of β1 integrin at both the mRNA and protein levels in Dcn^−/−^ and wild-type fibroblasts and these effects might influence adhesion and cell shape. The influence of DS decorin on vimentin offers a novel explanation for the reported delay in wound healing of Dcn^−/−^ mice and for the cutis laxa of EDS patients with defects in the biosynthesis of decorin.

## Supporting Information

Figure S1 **Influence of decorin and decorin protein core on protein expression by Dcn** ***^−/−^*** **fibroblasts analyzed.** 2D Gel electrophoresis of Dcn^−/−^ fibroblasts treated for 2 days with ascorbate-2-phosphate and decorin, decorin core and the respective control. Cells were harvested with a buffer containing 8 M urea, 2% (w/v) CHAPS, 20 mM DTT and subjected to isoelectric focusing between pH 3 and 10 according to manufactures’ instructions (ZOOM IPGRunner Kit, Invitrogen). (A) Representative 2D gel of the control after colloidal Coomassie staining. Marked square is magnified for control cells (B), decorin (C) and decorin core (D) treated cells. Gel spots (*) were analyzed by mass spectrometry (Center for Molecular Medicine, Cologne) and identified as vimentin.
(TIF)

Figure S2 **Vimentin mRNA expression in wild-type and Dcn^−/−^ fibroblasts measured at day 3 in culture.** Dcn^−/−^ fibroblasts were treated with decorin or decorin core or left untreated. Interestingly, wild-type fibroblasts express significantly more vimentin mRNA compared to Dcn^−/−^. This is supporting the reduced amount of vimentin protein found in the dermis of Dcn^−/−^ mice compared to wild-type. Furthermore, the core protein is not affecting the expression of vimentin in a 2D culture. Surprisingly the dermatan sulphate proteoglycan decorin significantly reduces the amount of vimentin mRNA at day 3 in culture (n = 3 independent experiments).
(TIF)

Figure S3 **Analysis of actin and fibrillar structures on the Dcn^−/−^ fibroblast surface after treatment with decorin or decorin core (core).** (A) Quantification of β-actin mRNA-expression with qRT-PCR (n = 3 independent experiments). CT values were normalized to reference genes as described in Materials & Methods. Students’ *t*-test (unpaired) showed no significant difference between the samples. (B) Immunofluorescence staining for F-actin with phalloidin coupled to Alexa 488 (green) in µ-slide VI. Nuclei were localized with DAPI (blue). Images show a merge of z-axis layers of the complete 3D matrix. (C) Quantification of F-actin immunofluorescence signal in merged layers normalized to the number of cells (nuclei) per image. Students’ *t*-test (unpaired) showed no significant differences between the samples. Data are expressed as mean ± SD (n = 3 independent experiments; for each condition 15 images were measured). (D) Atomic force microscopy (AFM) of the cortical actin microfilament network in Dcn^−/−^ fibroblasts at day 2.
(TIF)

Figure S4 **Partial colocalization of decorin and β1 integrin in 3D cultures of Dcn^−/−^ fibroblasts at day 6 treated with decorin and wild-type fibroblasts.** (A) Double immunofluorescence staining for β1 integrin (red) and decorin (green) in µ-slide VI of Dcn^−/−^ fibroblasts. Colocalizations of decorin and β1 integrin (yellow) are indicated by white arrowheads. (B) Wild-type fibroblasts fluorescence staining for β1 integrin (red) and decorin (green) in µ-slide VI. Nuclei were localized with DAPI (blue). Images show a merge of z-axis layers of the complete 3D matrix (digital magnification). (C) The representative Western blot for β1 integrin in the analyzed 3D wild-type fibroblast cultures and the respective control GAPDH (lower panel). (D) For quantification of β1 integrin expression grey-scale values of Western blot signal were normalized to GAPDH as loading control. Student’s *t*-test (unpaired) revealed a significant difference for decorin treated cells compared to the control. Data are expressed as mean ± SD (n = 3 independent experiments; *, p<0.05).
(TIF)

Figure S5 **Detection of β1 integrin** ***in vivo*** **in Dcn^−/−^ and wild-type newborn mouse dermis. (**A) Western blot for β1 integrin with dermis extracts of wild-type and Dcn^−/−^ mice (upper panel). Coomassie gel was used as loading control (lower panel). (B) Quantification of Western blot, β1 integrin signal was normalized to the Coomassie gel staining (n = 3 independent experiments; *, p<0.05).
(TIF)

Figure S6 **Detection of fibronectin and α5 integrin in Dcn^−/−^ fibroblasts.** (A) Western blot for α5 integrin (upper panel) and fibronectin (middle panel) of Dcn^−/−^ fibroblasts cultured for 6 days in the presence of ascorbate-2-phosphate and decorin or decorin core (core). The loading control GAPDH is shown in the lower panel. (B) Quantification of the Western blot for α5 integrin normalized to GAPDH (n = 3 independent experiments). (C) Quantification of the Western blot for fibronectin normalized to GAPDH (n = 3 independent experiments).
(TIF)
