# Supplementary figures and images for: The Dermatan Sulfate Proteoglycan Decorin Modulates α2β1 Integrin and the Vimentin Intermediate Filament System during Collagen Synthesis
Source: PLoS One. 2012 Dec 3;7(12):e50809. doi: 10.1371/journal.pone.0050809 (PMC3513320; doi:10.1371/journal.pone.0050809)

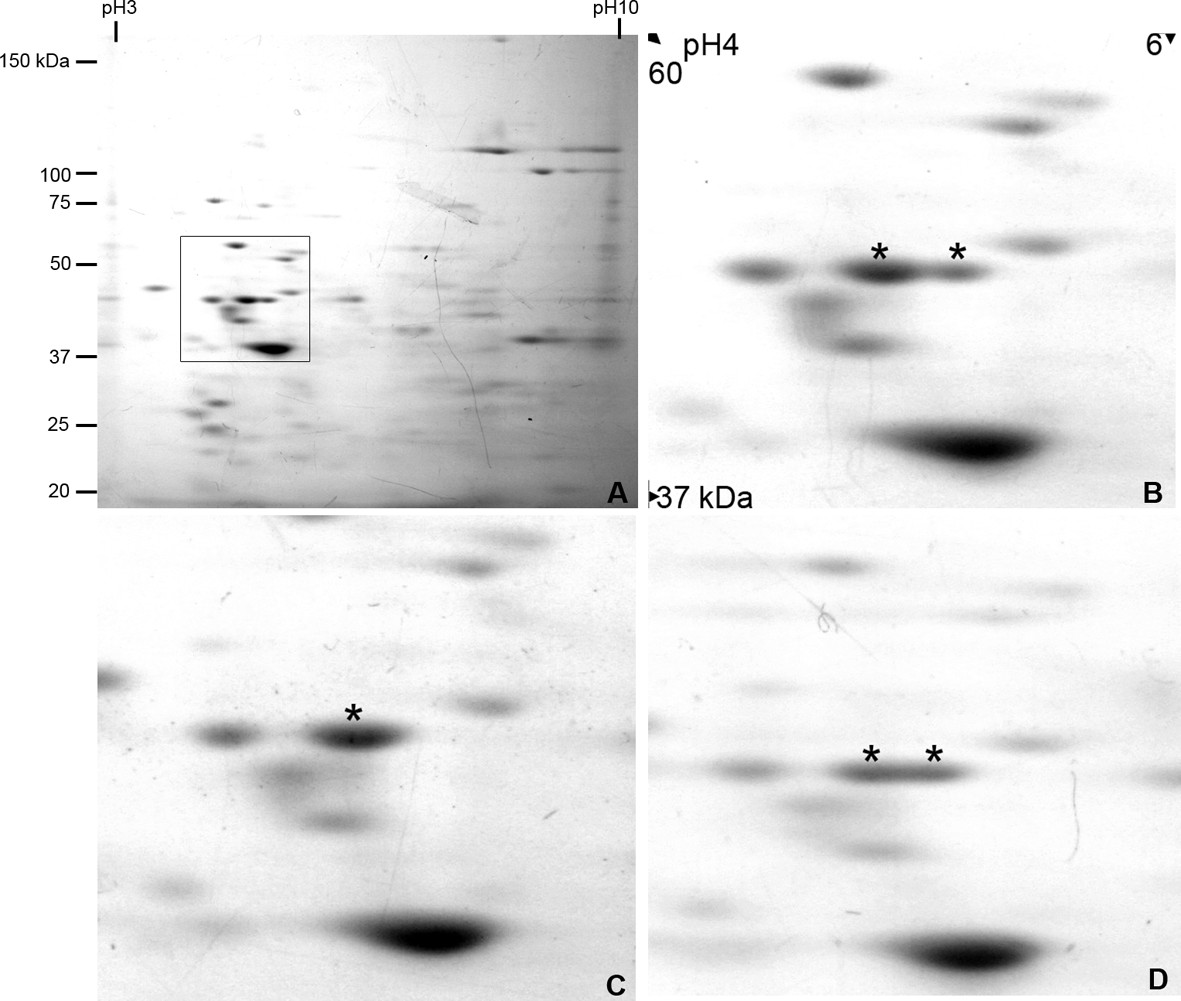

Supplement: Figure S1 — Influence of decorin and decorin protein core on protein expression by Dcn −/− fibroblasts analyzed. 2D Gel electrophoresis of Dcn−/− fibroblasts treated for 2 days with ascorbate-2-phosphate and decorin, decorin core and the respective control. Cells were harvested with a buffer containing 8 M urea, 2% (w/v) CHAPS, 20 mM DTT and subjected to isoelectric focusing between pH 3 and 10 according to manufactures’ instructions (ZOOM IPGRunner Kit, Invitrogen). (A) Representative 2D gel of the control after colloidal Coomassie staining. Marked square is magnified for control cells (B), decorin (C) and decorin core (D) treated cells. Gel spots (*) were analyzed by mass spectrometry (Center for Molecular Medicine, Cologne) and identified as vimentin. (TIF) [file pone.0050809.s001.tif]

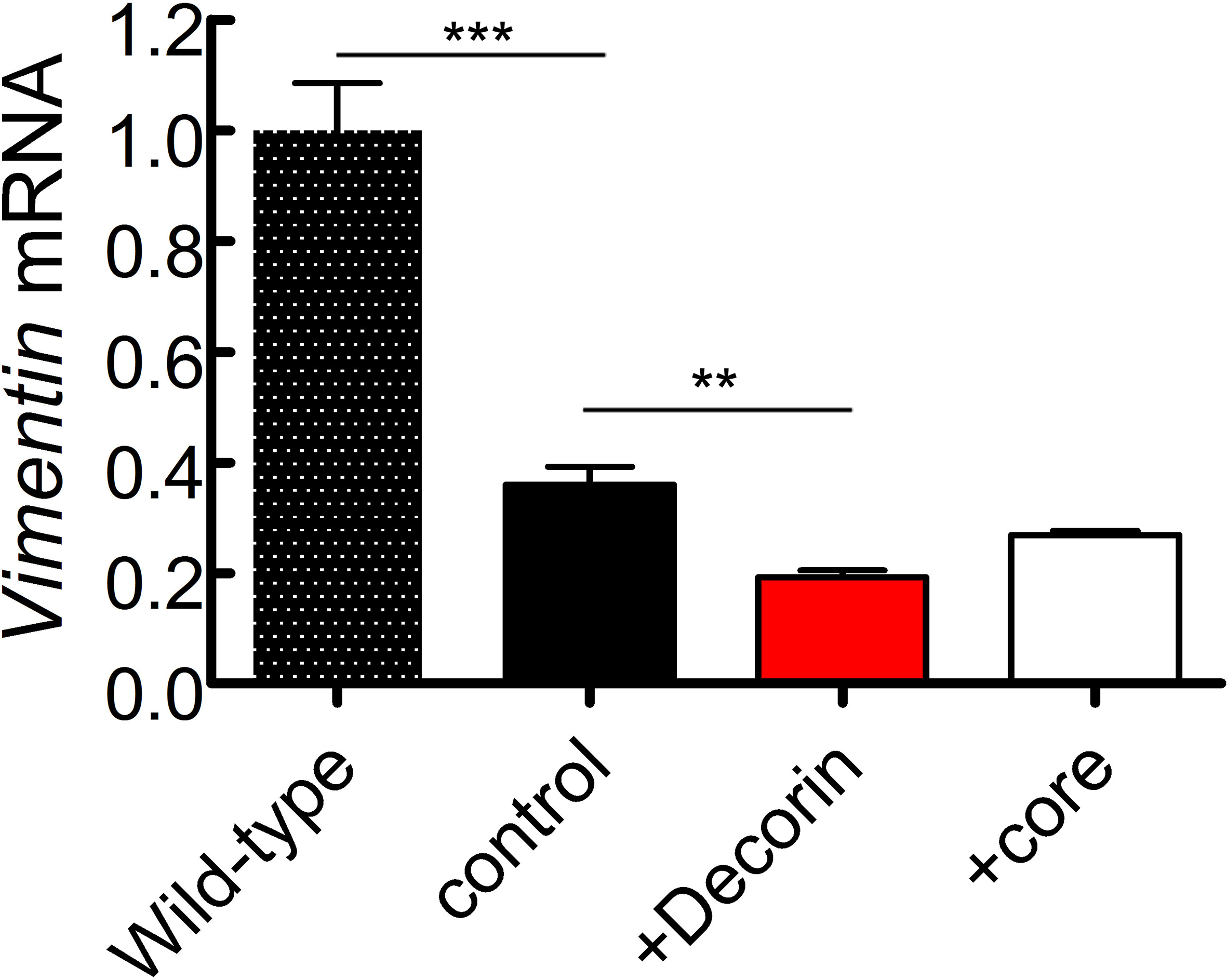

Supplement: Figure S2 — Vimentin mRNA expression in wild-type and Dcn−/− fibroblasts measured at day 3 in culture. Dcn−/− fibroblasts were treated with decorin or decorin core or left untreated. Interestingly, wild-type fibroblasts express significantly more vimentin mRNA compared to Dcn−/−. This is supporting the reduced amount of vimentin protein found in the dermis of Dcn−/− mice compared to wild-type. Furthermore, the core protein is not affecting the expression of vimentin in a 2D culture. Surprisingly the dermatan sulphate proteoglycan decorin significantly reduces the amount of vimentin mRNA at day 3 in culture (n = 3 independent experiments). (TIF) [file pone.0050809.s002.tif]

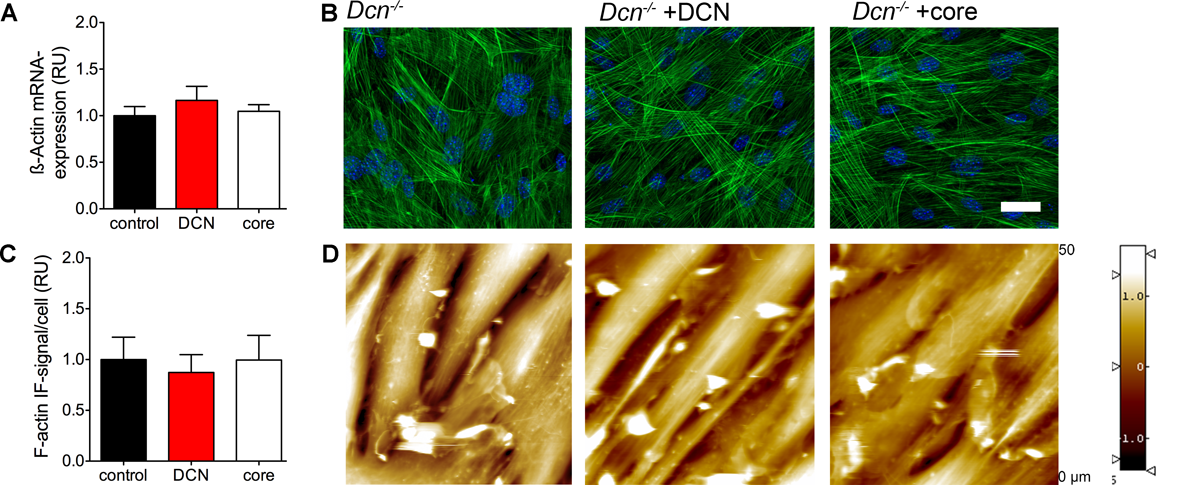

Supplement: Figure S3 — Analysis of actin and fibrillar structures on the Dcn−/− fibroblast surface after treatment with decorin or decorin core (core). (A) Quantification of β-actin mRNA-expression with qRT-PCR (n = 3 independent experiments). CT values were normalized to reference genes as described in Materials & Methods. Students’ t-test (unpaired) showed no significant difference between the samples. (B) Immunofluorescence staining for F-actin with phalloidin coupled to Alexa 488 (green) in µ-slide VI. Nuclei were localized with DAPI (blue). Images show a merge of z-axis layers of the complete 3D matrix. (C) Quantification of F-actin immunofluorescence signal in merged layers normalized to the number of cells (nuclei) per image. Students’ t-test (unpaired) showed no significant differences between the samples. Data are expressed as mean ± SD (n = 3 independent experiments; for each condition 15 images were measured). (D) Atomic force microscopy (AFM) of the cortical actin microfilament network in Dcn−/− fibroblasts at day 2. (TIF) [file pone.0050809.s003.tif]

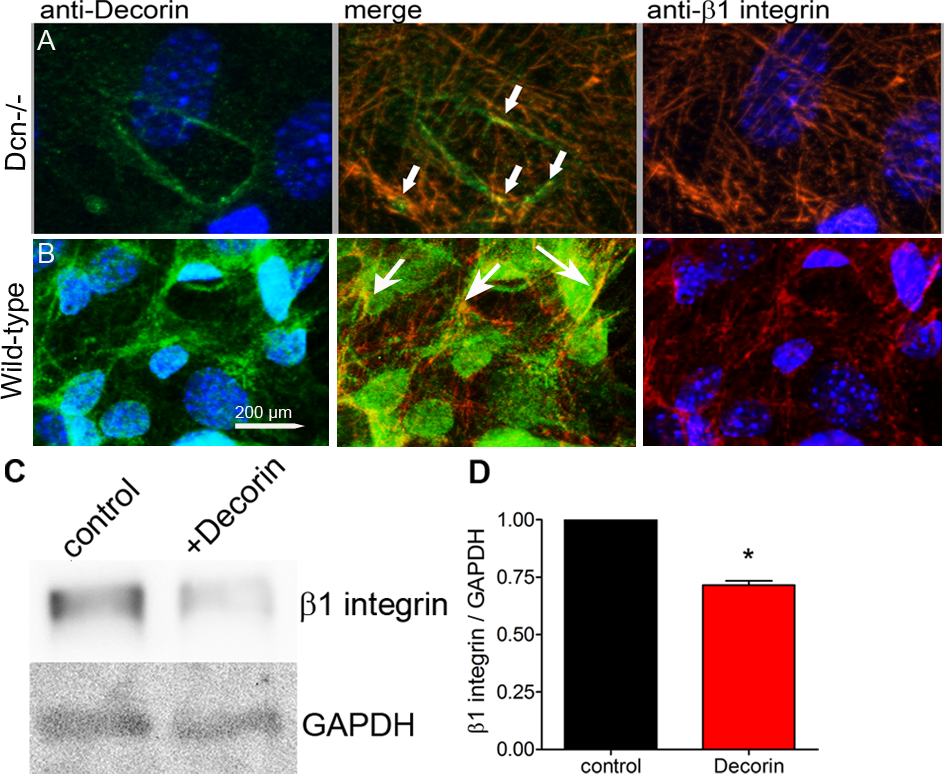

Supplement: Figure S4 — Partial colocalization of decorin and β1 integrin in 3D cultures of Dcn−/− fibroblasts at day 6 treated with decorin and wild-type fibroblasts. (A) Double immunofluorescence staining for β1 integrin (red) and decorin (green) in µ-slide VI of Dcn−/− fibroblasts. Colocalizations of decorin and β1 integrin (yellow) are indicated by white arrowheads. (B) Wild-type fibroblasts fluorescence staining for β1 integrin (red) and decorin (green) in µ-slide VI. Nuclei were localized with DAPI (blue). Images show a merge of z-axis layers of the complete 3D matrix (digital magnification). (C) The representative Western blot for β1 integrin in the analyzed 3D wild-type fibroblast cultures and the respective control GAPDH (lower panel). (D) For quantification of β1 integrin expression grey-scale values of Western blot signal were normalized to GAPDH as loading control. Student’s t-test (unpaired) revealed a significant difference for decorin treated cells compared to the control. Data are expressed as mean ± SD (n = 3 independent experiments; *, p<0.05). (TIF) [file pone.0050809.s004.tif]

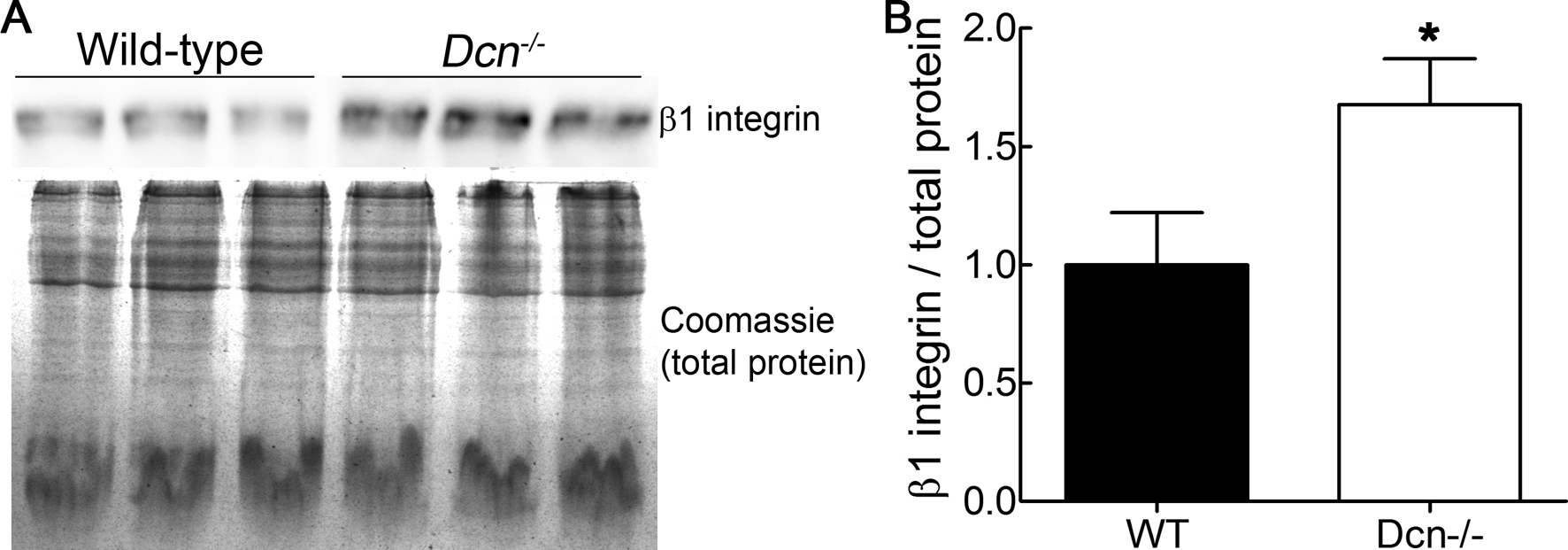

Supplement: Figure S5 — Detection of β1 integrin in vivo in Dcn−/− and wild-type newborn mouse dermis. (A) Western blot for β1 integrin with dermis extracts of wild-type and Dcn−/− mice (upper panel). Coomassie gel was used as loading control (lower panel). (B) Quantification of Western blot, β1 integrin signal was normalized to the Coomassie gel staining (n = 3 independent experiments; *, p<0.05). (TIF) [file pone.0050809.s005.tif]

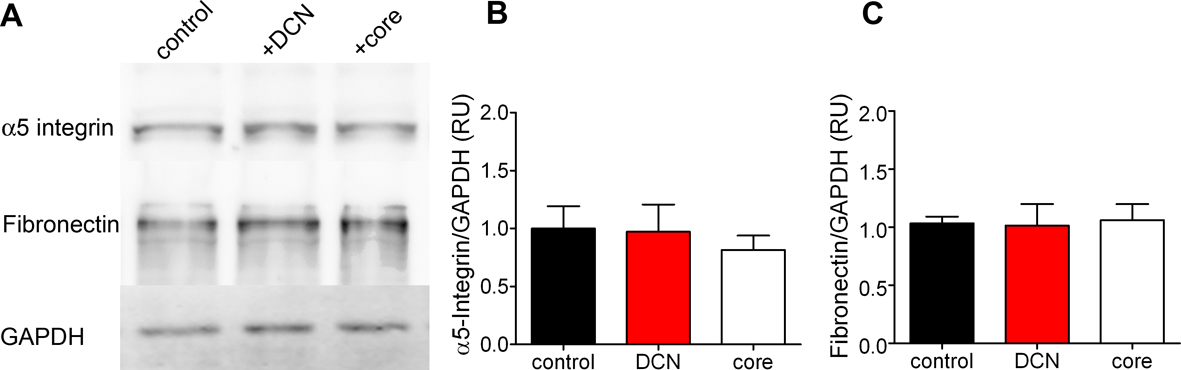

Supplement: Figure S6 — Detection of fibronectin and α5 integrin in Dcn−/− fibroblasts. (A) Western blot for α5 integrin (upper panel) and fibronectin (middle panel) of Dcn−/− fibroblasts cultured for 6 days in the presence of ascorbate-2-phosphate and decorin or decorin core (core). The loading control GAPDH is shown in the lower panel. (B) Quantification of the Western blot for α5 integrin normalized to GAPDH (n = 3 independent experiments). (C) Quantification of the Western blot for fibronectin normalized to GAPDH (n = 3 independent experiments). (TIF) [file pone.0050809.s006.tif]
